# The relationships between precarious employment, having a calling, and occupational well-being among young nurses: a cross-sectional study

**DOI:** 10.1186/s12913-024-11220-8

**Published:** 2024-06-24

**Authors:** Tanja Pesonen, Anu Nurmeksela, Marja Hult

**Affiliations:** 1 Department of Nursing Science, University of Eastern Finland, Kuopio, Finland; 2 Department of Sustainable Well-being, South-Eastern University of Applied Sciences, Mikkeli, Finland

**Keywords:** Calling, Occupational well-being, Precarious employment, Young nurses

## Abstract

**Background:**

Working in the healthcare sector seems less interesting than other sectors: the salary is low relative to the demands of the labour involved, and working conditions as well as management are perceived as poor. These factors may have an impact on the well-being of nurses in the healthcare sector. This study aims to explore the relationship between precarious employment and occupational well-being, in addition to the moderating effect of having a calling in this relationship among younger and older nurses.

**Methods:**

Cross-sectional survey data were collected among Finnish nurses (*n* = 5867) between October and November 2020. Data were collected on demographics, occupational well-being, precarious employment, and having a calling in the field. Multiple linear regression analyses were used to explore the associations.

**Results:**

Younger nurses perceived lower levels of occupational well-being and calling, and higher levels of precarious employment compared to older nurses. Precarious employment had a negative relationship with occupational well-being, and having a calling showed a positive relationship with regard to occupational well-being. No interaction effect of precarious employment and having a calling with occupational well-being was found.

**Conclusions:**

Young nurses’ occupational well-being, precarious employment, and calling should be studied further because they are in a weaker position in working life. Using a qualitative approach should be considered in order to obtain more in-depth information.

## Background

The global challenge of the availability of nursing staff and work commitment are more relevant today than ever [1]. According to estimates, there is currently a shortage of approximately four million nursing staff globally [2], and the aging of the nursing workforce is anticipated to worsen the situation. It is estimated that 4.7 million nurses will need to be educated and employed by 2030 in order to maintain the current workforce [3]. However, labouring in the social and healthcare sectors seems less attractive, especially from the point of view of young employees [4, 5]. The young employed generally experience lower job stability and higher job insecurity [3]. Additionally, due to low quality employment relationships, nursing can be identified as precarious work [6, 7].

Almost 30% of young nurses abandon the healthcare industry within the first few years as a result of unrealistic expectations about work and related factors [8, 9]. By focusing on occupational well-being, the retention and attractiveness of the healthcare sector can be strengthened [10, 11]. Nursing has traditionally been seen as a profession based on “having a calling” – an important component in trying to understand occupational well-being and general well-being in life [5, 12].

Precarious employment has been studied in sectors such as platform work and the hospitality industry, but there has been little research on precarious employment within the context of nursing [13]. There has also been no previous research on the relationship between precarious employment and occupational well-being among practical nurses and registered nurses. For this reason, it is important to study what kind of connections precarious employment has with occupational well-being and whether this perceived calling on the part of nurses shapes the connection between precarious employment and occupational well-being.

There is no unified definition for occupational well-being, which refers to psychological and physical factors as well as factors related to the work environment, including being able to cope on the job and occupational stress [14–16]. Promoting occupational well-being benefits organizations because it engages employees, helps create a positive work atmosphere, and improves the quality of care and patient satisfaction [10, 11]. Factors that increase the occupational well-being of nurses include meaningful work, the opportunity to learn, and the opportunity to influence and experience joy from the work done [9, 15]. Management style is also of great importance to an individual’s experience of occupational well-being [10, 11]. Other factors influencing occupational well-being include consideration of personal life situations, autonomy [4, 10], a sense of control over work, and relationships with other professionals [11, 17]. Studies show that young nurses experience more burnout, less work engagement, and have a higher intention to leave the occupation compared to older nurse [18, 19]. Moreover, they perceive the work environment more negatively and are unsatisfied with their individual expectations [20]. Based on these findings, we set the following hypothesis:

### Hypothesis 1

Younger nurses perceive lower levels of occupational well-being compared to older nurses.

There is no clear and well-established definition of precarious employment [21, 22], and it is regarded as a multidimensional phenomenon [13, 23]. The characteristic of precarious employment is the overall experience of uncertainty, which affects people’s emotional, psychological, and social well-being at work and outside of work [24]. Precarious employment is a combination of unstable employment [6, 19], having several potential employers [25, 26], low salary and lack of benefits [6, 22], or the lack of occupational rights and social equality [13, 27]. Precarious employment in healthcare is associated with heightened work demands, staff availability challenges, poor working conditions, low salary, and poor management [6, 13, 23].

Precarious employment is connected to lower occupational well-being and occupational safety [25, 28]. It can adversely affect employees’ physical and psychological health [6, 29], their social lives [24, 26], their quality of life [30, 31], and their perceived safety [32]. Irregular working hours and shift work increase the negative effects of precarious employment [6, 33]. Precarious employment especially affects young people [6, 26], women [13, 22], and employees with less work experience [6, 26]. For young employees, the negative relationship of precarious employment to occupational well-being appears as a factor related to health deterioration, exhaustion, and poor working conditions [34, 35]. Based on these findings, we hypothesized the following:

### Hypothesis 2

Precarious employment results in a negative relationship with occupational well-being.

A “calling” is often defined as a personal feeling [36, 37] that is prosocial in nature, involves work done for others [38, 39], and has received influences from outside the individual [40, 41]. A calling in nursing is described as a passionate inner motivation or a desire to help others by committing to nursing [36]. Having a calling has been found to increase nurses’ work motivation, job satisfaction, commitment to work, and their ability to cope with the demands of the job [5], even if there are challenges in the work environment [36, 39]. A calling can be seen as a source of occupational well-being and a meaningful and satisfying work experience [37]. Experiencing meaningfulness from work is an important component of a calling [12, 41]. Employees who feel called to their occupation feel more committed to their work. Based on research on having a calling at work, we formulated the following hypotheses:

### Hypothesis 3

Having a calling has a positive relationship with occupational well-being.

Given that precarious employment is expected to decrease and having a calling is expected to increase occupational well-being, we test their interaction, which is the major contribution of this study. The interplay between precarious employment and calling has not been analyzed before. This interplay is particularly interesting in the nursing profession, where both precarious employment and having a calling constitute significant factors. Therefore, we set the following hypothesis:

### Hypothesis 4

Having a calling moderates the relationship between precarious employment and occupational well-being.

## Methods

### Design

This was a cross-sectional survey conducted in the health and social care sector in Finland among practical nurses and registered nurses. The Strengthening the Reporting of Observational studies in Epidemiology [STROBE] guidelines for cross-sectional research were followed to report this study.

### Participants

Participants (practical nurses and registered nurses, subsequently referred to as nurses) were members of two Finnish trade unions for care workers and one company that provided temporary staff in the care sector (*n* = 93,254). The survey data were collected online using the Webropol tool between September and November 2020. Those in charge of the human resources services of both the trade unions and the personnel service company were contacted, and they sent an announcement about the study by email to their members or as part of a monthly membership letter.

A total of 7,925 persons responded to the survey, achieving a response rate of 8.5%. Seven respondents were excluded because they did not give their informed consent. One respondent was excluded because their age exceeded 74, which was the inclusion criterion for the study. One respondent was also excluded because the number of missing values exceeded 50%. Furthermore, this study focused only on practical nurses and registered nurses, and after excluding other professionals (*n* = 2,049), the final sample size was 5,867 respondents. In the questionnaire, the background variables were age, gender, education, working sector, profession, and employment contract.

### Data collection

#### The work experience measurement scale (WEMS)

Occupational well-being was measured using the Work Experience Measurement Scale [WEMS; 42]. The scale contains 32 statements divided into six subscales: supportive working conditions (seven statements, a sample statement: “We encourage and support each other at work”), internal work experiences (six statements, a sample: “I am happy when I go to work”), autonomy (four statements, a sample: “I decide when to do the various work tasks”), time experience (three statements, a sample: “I do not need to work more than my scheduled hours”), leadership (six statements, a sample: “My boss is available when I need him/her”), and process of change (six statements, a sample: “The process of change was done with an open dialogue”) [43]. Statements were answered on a six-point Likert scale, with options ranging from “totally agree (6)” to “totally disagree (1).” A total score was the mean of subscales, with a higher score indicating better occupational well-being. The data concerning all the questions of WEMS contained 0.2–2.1% missing values. Previously, a Cronbach’s alpha of 0.94 was reported among care workers [44].

### Employment precariousness scale (EPRES)

Precarious employment was measured using the Employment Precariousness Scale [EPRES; 45]. The scale contained 22 questions divided into six subscales: temporariness (two statements, such as “How long is your current employment contract valid?”), wages (three statements, such as “Approximately how much do you earn per month after taxes?”), disempowerment (three statements, such as “How were your working hours settled for your current job?”), vulnerability (six statements, such as “Indicate how often You are defenseless towards unfair treatment by your superiors”), rights (seven statements, such as “Do you have the right to parental leave?”), and exercise of rights (five statements, such as “How often, in the organization where you work, are you able to take the weekend off/weekly rest without problem?”). The responses were converted to a scale of 0–4. Value (4) represents high employment precariousness, and value (0) represents low employment precariousness. Means were calculated for each subscale, and the total score was the mean of all the subscales. EPRES contained 0.1‒0.7% missing values.

### Calling and vocation questionnaire (CVQ)

The multidimensional Calling and Vocation Questionnaire [CVQ; 46] was used to measure the degree to which participants reported perceiving their job as a calling. The CVQ includes 24 statements divided into two dimensions of a calling: CVQ Search, which evaluates a search for one’s calling, and CVQ Presence, which assesses a current calling [47]. This study used the CVQ Presence subscale with 12 statements divided into three dimensions: transcendent summons (a sample statement: “I was drawn by something beyond myself to pursue my current line of work”), purposeful work (a sample statement: “I see my career as a path to purpose in life”) and prosocial orientation (a sample statement: “I am always trying to evaluate how beneficial my work is to others”). Response options ranged from ‘(1) not at all true of me’ to ‘(4) absolutely true of me.’ A total score was the mean of all items, and a higher score indicated a higher level of calling. The data regarding CVQ contained 0.1% missing values. An earlier study among care workers reported an internal consistency reliability of Cronbach’s α = 0.86 [46].

The WEMS, EPRES, and CVQ measures have previously been translated from English to Finnish and “backtranslated” by a professional translator. Thereafter, the questionnaire was tested with 10 nurses and nursing researchers before data collection commenced.

### Data analysis

For the analysis, the respondents were divided into two groups based on age: ≤ 35 (later called younger) and > 35 (later called older). Statistical differences were assessed using the χ^2^ test, with statistical significance set to *p* ≤ 0.05. Independent sample *t*-tests and one-way analysis of variances (ANOVA) were used to detect differences between study variables and respondents’ ages and characteristics. Pearson’s correlation coefficient was used to examine the correlations between variables (WEMS, EPRES, and CVQ). Multiple linear regression analyses were used to test the research hypotheses and examine the relationships between age, precarious employment, a calling, and occupational well-being. We ran three models to examine these relationships. First, an unadjusted model was used to produce a bivariate association of occupational well-being with all the variables separately. Second, all the variables were adjusted for sociodemographic factors (gender, education, working sector) in Model (1) Finally, an interaction of precarious employment and having a calling was examined in Model (2) SPSS 27.0 was used for the data analysis.

## Results

Table 1 shows that there were 5,867 respondents with a mean age of 48.3 (range 19–74) years; 13.9% were under 35 years old and 84.5% over 35 years old. Most of the respondents (93%) were practical nurses, and 7% were registered nurses. The respondents worked in healthcare (46%), social services (33%), and early education and childcare (20%). Younger workers were registered nurses more frequently than older ones, had lower levels of education, and worked more frequently with temporary employment contracts.

**Table 1:** Characteristics of respondents divided into age groups

	≤ 35 (years) *n* (%)	> 35 (years) *n* (%)	*p*
Gender			0.656
Men	34 (4.2%)	226 (4.6%)	
Women	769 (95.8%)	4701 (95.4%)	
Education			< 0.001
Elementary school	5 (0.6%)	57 (1.2%)	
Upper secondary level	709 (87.7%)	4013 (81.1%)	
Bachelor’s degree	90 (11.1%)	832 (16.8%)	
Master’s degree or higher	4 (0.5%)	45 (0.9%)	
Working sector			0.115
Healthcare	400 (49.1%)	2234 (45.3%)	
Social services	252 (31.0%)	1683 (34.1%)	
Early education and childcare	162 (19.9%)	1019 (20.6%)	
Profession			0.003
Practical nurse	747 (91.7%)	4618 (93.1%)	
Registered nurse	68 (8.3%)	342 (6.9%)	
Employment contract			< 0.001
Permanent	588 (72.7%)	4248 (86.4%)	
Temporary	221 (27.3%)	669 (13.6%)	

*Note: p*-value obtained using *t*-test and chi-squared tests

Table 2 shows the scores for occupational well-being (measured with WEMS), precarious employment (measured with EPRES), and having a calling (measured with CVQ) for younger (≤ 35) and older (> 35) nurses. Older nurses had significantly higher scores for occupational well-being, except for the subscale *process of change*, which showed no difference between age groups. The *internal work experience* subscale showed the highest scores for both groups. Younger nurses perceived significantly higher employment precariousness in every dimension. The *rights* subscale showed the highest scores for younger nurses. The *wages* subscale displayed remarkably similar values for both groups. The total score for calling was significantly (*p* < 0.001) higher among older nurses. The *transcendent summons* subscale, however, showed no difference between the age groups. Precarious employment was higher and calling as well as occupational well-being were lower among younger nurses.

**Table 2:** Means and standard deviations (SD) for dimensions of WEMS, EPRES, and CVQ by age group

	≤ 35 (years) Mean (SD)	> 35 (years) Mean (SD)	*p*
WEMS total (scale 1–6)	3.52 (0.84)	3.67 (0.84)	< 0.001
Working conditions	3.67 (0.74)	3.83 (0.71)	< 0.001
Internal work experience	4.33 (0.97)	4.56 (0.97)	< 0.001
Autonomy	3.18 (1.14)	3.40 (1.19)	< 0.001
Time experience	3.37 (1.29)	3.47 (1.28)	0.024
Leadership	3.55 (1.25)	3.73 (1.25)	< 0.001
Process of change	3.03 (1.22)	3.02 (1.26)	0.873
EPRES total (scale 0‒4)	1.37 (0.49)	1.05 (0.46)	< 0.001
Temporariness	0.85 (0.90)	0.42 (0.71)	< 0.001
Wages	1.79 (0.76)	1.71 (0.77)	0.003
Disempowerment	0.41 (0.77)	0.26 (0.61)	< 0.001
Vulnerability	1.70 (0.96)	1.42 (0.91)	< 0.001
Rights	2.08 (1.16)	1.23 (1.04)	< 0.001
Exercise of rights	1.40 (0.80)	1.26 (0.81)	< 0.001
CVQ total (scale 1‒4)	2.54 (0.59)	2.63 (0.56)	< 0.001
Transcendent summons	2.38 (0.60)	2.41 (0.58)	0.113
Prosocial orientation	2.93 (0.67)	2.98 (0.65)	0.033
Purposeful work	2.32 (0.78)	2.49 (0.75)	< 0.001

Note: WEMS: Work experience measurement scale, EPRES: Employment precariousness scale, CVQ: Calling and vocation questionnaire. *p*-value from t-tests

Table 3 shows that the internal consistency of the measures was quite acceptable; only the internal consistency of the measure of precarious employment was lower than the other measures. Correlations among the study variables were small to medium (Table 3).

**Table 3:** Cronbach’s αs (in parentheses) and Pearson’s correlations for the study variables

	WEMS	EPRES	CVQ
WEMS	(0.94)		
EPRES	-0.45**	(0.44)	
CVQ	0.45**	-0.13**	(0.85)

Note: WEMS: Work Experience Measurement Scale, EPRES: Employment Precariousness Scale, CVQ: Calling and Vocation Questionnaire. ** Correlation is significant at the 0.01 level (2-tailed)

Table 4 shows the results of the multiple linear regressions. In bivariate analyses, occupational well-being was significantly positively associated with higher age and having a calling, and significantly negatively associated with precarious employment. In the multivariate regression, younger age and precarious employment were negatively associated with occupational well-being, while having a calling was positively associated with occupational well-being. These findings support Hypotheses 1, 2 and 3. This model explained 39.4% (R² adjusted) of the variation in occupational well-being. Finally, when testing the two-way interaction (precarious employment × calling), no significant interaction was found. Thus, Hypothesis 4 is rejected.

**Table 4:** Associations between younger age, precarious employment, having a calling, and occupational well-being among nurses

	β	95% CI
**Model 0**		
Younger age^a^	0.156***	0.097‒0.215
Precarious employment	-0.797***	-0.837‒-0.758
Calling	0.700***	0.667‒0.733
**Model 1**		
Younger age^a^	-0.123***	-0.170‒-0.075
Precarious employment	-0.710***	-0.746‒-0.673
Calling	0.619***	0.589‒0.649
*R* ^*2*^	*0.394*	
**Model 2**		
Younger age^a^	-0.122***	-0.170‒-0.074
Precarious employment	-0.620***	-0.783‒-0.458
Calling	0.658***	0.583‒0.733
Precarious employment × Calling	-0.034^ns^	-0.094‒0.026
*R* ^*2*^	*0.394*	

Note: Model 0 = Bivariate associations, unadjusted estimates, Model 1 = Adjusted for younger age, precarious employment, calling, gender, education, and working sector, Model 2 = Model 1 + interaction, ^a^Ref = Older age, *β* = Unstandardized beta, CI = Confidence interval, *** *p* < 0.001, ^ns^non-significant

## Discussion

This study investigated the relationship between precarious employment and occupational well-being, as well as the moderating effects of having a calling on this relationship. To the best of our knowledge, this is the first study to examine this topic from this perspective in nursing. Although precarious employment was associated with decreased occupational well-being and having a calling was associated with increased occupational well-being, their interaction was not significant. This means that having a calling did not alleviate the detrimental effect of precarious employment on occupational well-being in this sample of nurses.

The results confirmed the negative relationship between precarious employment and occupational well-being, as was hypothesized. This is in line with previous literature indicating that indecent and unstable work is connected to lower occupational well-being [47], and it influences the mental and physical health of employees, increases workload, and worsens working conditions [6]. Previous studies have found that precarious employment affects specifically young workers [22, 26]. The results of this study add to the body of evidence on the weaker position of young workers in the labor market, including in healthcare workplaces.

Low salaries and temporary employment contracts have been one of the main features of precarious employment [6, 23]. Accordingly, we showed that, for young nurses, the lack of rights was the main source of precariousness. Usually, rights are defined by laws, regulations, and collective agreements [35, 48]. It may be that young nurses do not necessarily know their rights, so they do not know how to demand them. Low salaries and the experience of vulnerability were also features of precarious employment for young nurses. In Finland, wages are primarily negotiated on the collective level. Despite this, nurses’ salaries are low compared to other professional groups. Salary is not perceived to correspond to the demands of the work, which may increase feelings of unfairness. The experience of vulnerability among young nurses may be due to poor working conditions (e.g., workload, poor work environment, and unsafe workplace), unfair treatment, or poor management [48]. In future research, young nurses’ experiences of vulnerability should be examined in relation to the resources and competence of nursing management. Despite experiences of vulnerability, one dimension of precarious employment—temporariness—can also be connected to a sense of freedom and flexibility [28], especially for employees who work voluntarily in fixed-term employment relationships [22]. Therefore, undertaking fixed-term work may also increase occupational well-being. Future studies should examine this phenomenon further.

The results showed a positive relationship between having a calling and occupational well-being, confirming the study hypothesis. Motivation to work for other people in the perception of having a calling became the most important dimensions for younger nurses, and meaningful work as well as the motivation to help others for older nurses. The results are in line with previous studies that proposed that perceiving work as important and a person’s own motivation increase the feeling of having a calling [12], thereby improving occupational well-being [49].

Previous literature indicates that for young nurses, meaningfulness and having a calling are important factors that increase occupational well-being [7, 14]. In the current study, younger nurses experienced lower feelings of having a calling. In fact, having a calling has been found to be stronger among older nurses [5]. Young nurses may have different kinds of values related to work, and they seem to be less committed to work than older nurses [9]. In addition, young nurses seek to receive recognition for their work and adequate compensation for the work done [36]. These could be the reasons why young nurses do not have a greater tendency to have a calling, but when they do, the calling may be the reason for their better occupational well-being. Overall, in this study, younger nurses had lower levels of having a calling, higher perceived employment precariousness, and lower occupational well-being, as was hypothesized based on previous studies.

This study showed no moderating effect of calling on the negative association between precarious employment on occupational well-being, thus rejecting the fourth hypothesis. However, according to the previous studies, calling might be a factor that mitigates the negative effect of precarious employment on occupational well-being because it has shown to increase the ability cope with the demands of job in the challenging work environment [5, 36, 39]. This relationship needs more in-depth investigation.

### Strengths and limitations

This study has a few limitations that should be acknowledged. First, this study used a cross-sectional design, so it was not possible to draw causal conclusions. Future studies should use a longitudinal design to explore the relationships between precarious employment, having a calling, and occupational well-being. Additionally, studies with a qualitative approach may provide a deeper understanding of how having a calling affects the relationship between precarious employment and occupational well-being, in addition to how age influences this. The strength of this study is that we achieved a good number of respondents, even though the response rate was small (8.5%). However, due to the low response rate, the results should be treated with caution.

The employment precariousness scale (EPRES) received low internal consistency in this study which can be considered low compared to previous studies [50]. This is most likely due to the target group of the study. First, the frequency of temporary contracts is low. Second, the disempowerment of healthcare workers is minimal because a large number of Finnish care workers are unionized. This indicates that their salaries and working hours, for example, are negotiated through collective agreements. Therefore, future studies should modify the measure to be better suited to the healthcare context.

## Conclusion

To conclude, having a calling was found to be positively connected to occupational well-being among nurses, and precarious employment was negatively connected. The results can be utilised to identify problems related to the occupational well-being of nurses of different ages and how they influence their occupational well-being. The information obtained from the study can also be applied to the development of nursing management. In the future, young nurses’ occupational well-being, precarious employment, and having a calling should be studied further by incorporating, for example, a qualitative approach in order to obtain more in-depth information.

## Data Availability

The datasets analysed during the current study are available from the corresponding author on reasonable request.
